# Exophthalmic Goitre

**Published:** 1887-12

**Authors:** 


### EXOPHTHALMIC GOITRE.

In a case of exophthalmic goitre recently reported by Jendrassik, beside the cardinal symptoms of exophthalmos, goitre, and tachycardia, together with tremor and restlessness, there were also noted paralysis of the ocular muscles, of the facial and masticatory muscles, and, to a lesser degree, of the muscles of the pharynx and of the upper extremity. The writer satisfied himself that this paralysis was nuclear, and not peripheral — an external ophthalmoplegia, due to disease of the nuclei in the medulla, a polioencephalitis superior (Wernicke), or, as he prefers to call it, poliomyelencephalitis superior. He questions whether the presence of the symptoms of exophthalmic goitre and of ophthalmoplegia are due to a mere coincidence. He cites other cases like the one reported, and suggests that exophthalmic goitre may be due to disease of the medulla or mesencephalon. Since three distinct and distant parts of the body are involved, the lesion must be located in some place where the different efferent nerves lie near one another. He contests the theories that exophthalmic goitre is a general neurosis, or a lesion of the cervical sympathetic, and claims that it is due to a central lesion, located in the medulla or mesencephalon. The fact that only one or two of the three cardinal symptoms may be present is explained by supposing that only one or two nuclei are involved. Various cases of exophthalmic goitre show, moreover, that there are not infrequently symptoms present which point to a bulbar lesion — opthalmoplegia, diabetes mellitus, etc. — and, in other cases, the existence of muscular atrophy shows that the nuclei in the cords are also affected. The writer, therefore, believes that in this disease there is a constant and circumscribed lesion of the gray matter in the medulla, about at the level of the nucleus of the facial. This theory is further corroborated by the experiments of Filehne, who found that injury of the anterior fourth of the restiform body was followed by the three cardinal symptoms of exophthalmic goitre.—*Boston Medical and Surgical Journal.*

